# Is *MTHFR* 677 C>T Polymorphism Clinically Important in Polycystic Ovarian Syndrome (PCOS)? A Case-Control Study, Meta-Analysis and Trial Sequential Analysis

**DOI:** 10.1371/journal.pone.0151510

**Published:** 2016-03-16

**Authors:** S. Justin Carlus, Saumya Sarkar, Sandeep Kumar Bansal, Vertika Singh, Kiran Singh, Rajesh Kumar Jha, Nirmala Sadasivam, Sri Revathy Sadasivam, P. S. Gireesha, Kumarasamy Thangaraj, Singh Rajender

**Affiliations:** 1 CSIR-Centre for Cellular and Molecular Biology, Hyderabad, India; 2 Centre for Genetics and Inherited Diseases (CGID), Taibah University, Al- Madinah, Kingdom of Saudi Arabia; 3 Division of Endocrinology, CSIR-Central Drug Research Institute, Lucknow, India; 4 Banaras Hindu University, Varanasi, India; 5 Maaruthi Medical Centre and Hospitals, Erode, Tamil Nadu, India; 6 Yogi Vemana University, Kadapa, Andhra Pradesh, India; University of Texas School of Public Health, UNITED STATES

## Abstract

**Background:**

Optimum efficiency of the folate pathway is considered essential for adequate ovarian function. 677 C>T substitution in the 5, 10-methylene tertrahydrofolatereductase (*MTHFR*) gene compromises activity of the MTHFR enzyme by about 50%. The significance of correlation between 677C>T substitution and PCOS remains dubious due to the low power of published studies.

**Methods and Results:**

We analyzed *MTHFR* 677 C>T site in ethnically two different PCOS case-control groups (total 261 cases and 256 controls) from India. The data analysis revealed a lack of association between this polymorphism and PCOS [OR = 1.11 (95%CI = 0.71–1.72), P = 0.66]. Group-wise analysis on the basis of ethnicity also revealed no association in any of the ethnic groups [Indo-Europeans, P = 1; Dravidians, P = 0.70]. Homocysteine levels did not differ significantly between cases (15.51 μmol/L, SD = 2.89) and controls (15.89 μmol/L, SD = 2.23). We also undertook a meta-analysis on 960 cases and 1028 controls, which suggested a significant association of the substitution with PCOS in the dominant model of analysis (OR = 1.47 (95%CI = 1.04–2.09), P = 0.032]. Trial sequential analysis corroborated findings of the traditional meta-analysis. However, we found that the conclusions of meta-analysis were strongly influenced by studies that deviated from the Hardy Weinberg equilibrium. A careful investigation of each study and a trial sequential analysis suggested that 677 C>T substitution holds no clinical significance in PCOS in most of the populations.

**Conclusion:**

In conclusion, *MTHFR* 677 C>T polymorphism does not affect PCOS risk in India. The association seen in the meta-analysis is due to an outlier study and studies showing deviation from the Hardy Weinberg equilibrium.

## Introduction

Polycystic ovarian syndrome (PCOS) is the most widespread form of female infertility. Quoted prevalence of PCOS ranges from 5 to 25% [1–2]. The hormonal condition in polycystic women presents an excessive production of androgens, which disturbs menstrual cycle and ovulation, leading to primary or secondary infertility. This problem impairs female fertility to varying degrees and affects approximately 10% of women in India, but the incidence is on a sharp rise [3,4]. Around 4% of unselected population of reproductive age and 7% of the Caucasian population is believed to have this syndrome [5]. PCOS shows a heterogeneous mode of presentation characterized by metabolic disorder with anovulation, chronic anovulatory cycles, oligomenorrhea, endometrial dysplasia, non-insulin dependent diabetes mellitus (Type II), dyslipidaemia (hyper), hypertension, sub-fertility and enlarged cystic ovaries [6]. In 2003, the Rotterdam consensus of the American Society for Reproductive Medicine/ European Society of Human Reproduction and Embryology stated that polycystic ovaries, oligo/anovulation and hyperandrogenism are the most detectable traits of PCOS [7].

Etiology of PCOS remains largely unknown; however, the syndrome is thought to be multi-factorial with hyperinsulinemia and lifestyle factors being the prominent contributing factors. Questions about the possible effect of folic acid on ovarian function were raised in the late 1960s, with the finding that deficiency/excess of folates partially inhibited ovulation in immature super-ovulated rats [8]. Later, a research group demonstrated that ovarian biopsies from folate deprived monkeys showed degeneration of Graffian follicles with an increase in atretic and cystic follicles, accompanied by a depletion of granulosa cells and reduction or even absence of corpora lutea [9]. The above suggested a critical importance of one carbon metabolism in ovarian function and dysfunction. Since folate cycle and homocysteine-methionine cycle run in conjugation, alterations in the folate cycle may disturb homocysteine-methionine balance and maintenance of the methyl pool [10]. There is now sufficient evidence supporting high homocysteine levels in PCOS cases [11–13]. Interestingly, homocysteine level returned to normal following folic acid supplementation [13]. This makes methylenetetrahydrofolate reductase (*MTHFR*) an important gene for investigation in PCOS as decreased efficiency of folate/homocysteine pathway could increase the risk.

It has been experimentally shown that a functional substitution, 677 C>T (alanine is substituted by valine, rs1801133), in the *MTHFR* gene compromises activity of this gene and the folate pathway by about 50% [14–16]. There are reports of a significantly elevated PCOS risk associated with mutant genotypes at this locus [17–18]. On the other hand, a higher frequency of the supposed risk allele (T) has been reported in controls [19–20]. Most of the studies that negate a correlation between 677 C>T and PCOS severely lack statistical power required to support the inference [19–25]. Apart from a few case-control studies based on small sample sizes, at least three meta-analyses have previously investigated the importance of this polymorphism in PCOS [26–28]. We undertook the present case-control study on a larger set of samples so as to draw conclusions with adequate power. Further, we recruited two ethnically different case-control groups to evaluate significance of ethnic variations on the correlation. We also undertook a trail sequential analysis to critically examine the inference and power of the study and validate the findings of the traditional meta-analysis.

## Materials and Methods

### Study population

This study was approved by the Institutional Human Ethics Committee of the Maaruthi Medical Centre and Hospitals, Erode, Tamil Nadu and that of the CSIR-Central Drug Research Institute, Lucknow, Uttar Pardesh. We calculated the sample size using G power calculator, assuming a 10% frequency of the polymorphism (already reported in Indian populations), p = 0.05, study power of 90% and effect size of 0.3. This suggested a sample size of 234 (117 cases and 117 controls). We recruited 517 subjects with or without PCOS from the Maaruthi Medical Center and Hospitals, Erode, Tamil Nadu and the Institute of Medical Sciences, Banaras Hindu University, Varanasi. Relevant details about the subjects such as age, height, weight and age at marriage were noted. The inclusion criteria of patients were based on the Rotterdam Revised 2003 (2 out of 3) diagnosis guidelines. This included the presence of two out of the following three features (after the exclusion of related disorders): (1) Oligo- or an-ovulation, (2) Clinical and/or biochemical signs of hyperandrogenism, and (3) Polycystic ovaries. Hyper-androgenism was diagnosed by measuring serum testosterone, androstenedione, and DHEAS (Dehyroepiandrosteronesulphate) levels in the early follicular phase and by looking at the pattern and extent of terminal hair growth (hirsutism). Menstrual history of the patients was recorded to diagnose oligo-/ameno-rrhea. Ovarian morphology was studied by transvaginal ultrasonography to find if enlarged ovaries with at least 12 peripherally arranged immature follicles, a characteristic feature of PCOS, were seen.

We recruited 261 patients (168 from the Dravidian population and 93 from the Indo-European population) following the above inclusion criteria. The exclusion criteria consisted of the presence of congenital adrenal hyperplasia, Cushing’s syndrome, thyroid dysfunction or hyperprolactinemia. The patients were categorized in obese and non-obese groups according to the World Health Organization (WHO) criteria. Women having BMI in the range of 27.3–32.3 (overweight) and >32.3 (obese) were included in the obese group while women having BMI in the range of 19.1–25.8 (Normal) and 25.8–27.3 (Marginally overweight) were included in the non-obese group [29]. The BMI values for the Indo-European subjects could not be collected; therefore, this classification was applied to the Dravidian group only. Accordingly, in the Dravidian patient group, 144/169 (85.2%) fell in the obese and 25/169 (14.8%) fell in the non-obese category. The BMI in the hyperandrogenic group (36.91±5.18) was significantly higher in comparison to the normoandrogenic (29.39±4.0) group (P = 0.0001).

Two hundred fifty six ethnically matched control samples (comprising of 156 subjects from the Dravidian population and 100 from the Indo-European population) were collected from proven fertile women who volunteered to participate in the study. The controls were recruited according to the defined inclusion criteria of proven fertility with normal menstrual cycle and ovarian morphology, but no history of sub-fertility treatment. Peripheral blood samples (3–5 ml) of the patients and controls were collected for genetic analysis. Informed written consents were obtained from all participants. Homocysteine level in serum samples was measured using an enzymatic test based kit manufactured by Diazyme Laboratories (USA).

### DNA isolation

Genomic DNA was isolated from peripheral blood samples according to the protocol described in our previous study [30]. DNA concentration was determined using a spectrophotometric method by reading absorbance at 260 nm, followed by dilution to 10 ng (working concentration) in the standard TE buffer. The quality of DNA preparation was evaluated on 1% agarose gel.

### PCR amplification and genetic analyses

677C>T region of the *MTHFR* gene was amplified using primers and the protocol published in our earlier study [31]. Briefly, PCR was carried out in a reaction volume of 10 μl each in thin walled tubes, consisting of 1.0 μl of PCR buffer (10X), 1.0 μl of MgCl_2_ (25 mM), 1.0 μl of dNTPs (10 mM), 2.0 pM of each of the forward (5’ CATCCCTATTGGCAGGTTACCC 3’) and reverse (5’ GGGAAGAACTCAGCGAACTCAG 3’) primers, 1.0 unit of Taq DNA polymerase enzyme (Applied Biosystems) and 40 ng of genomic DNA. PCR amplification was carried out using the ABI Veriti thermal cycler (Applied Biosystems, USA) with the following conditions; denaturation at 95°C for 10 minutes followed by 35 cycles of denaturation at 95°C for 30 seconds, annealing at 63°C for 30 seconds and polymerization at 72°C for 40 sec, and a final stage of polymerization at 72°C for 7 minutes. The amplicons were directly sequenced using BigDye^TM^ chain termination chemistry on the ABI 3730 DNA analyzer (Applied Biosystems, USA)[32]. Multiple alignment and sequence analyses were undertaken using the AutoAssembler Software (Applied Biosystems, USA).

### Statistical analyses

Genotype data for the control group were analyzed for fitness in the Hardy Weinberg (HW) equilibrium. All statistical analyses were performed using the SPSS software (www.spss.com). The frequency of the major and minor alleles was compared between cases and controls using the chi-square (χ^2^) test. Pooled data for all cases (Indo-European and Dravidian) were compared with controls, followed by the comparison of cases and controls within each group. The two-sided P value of less than 0.05 (95% level of confidence) was considered significant for statistical inference.

### Meta Analysis

#### Literature search

**Fig 1:**
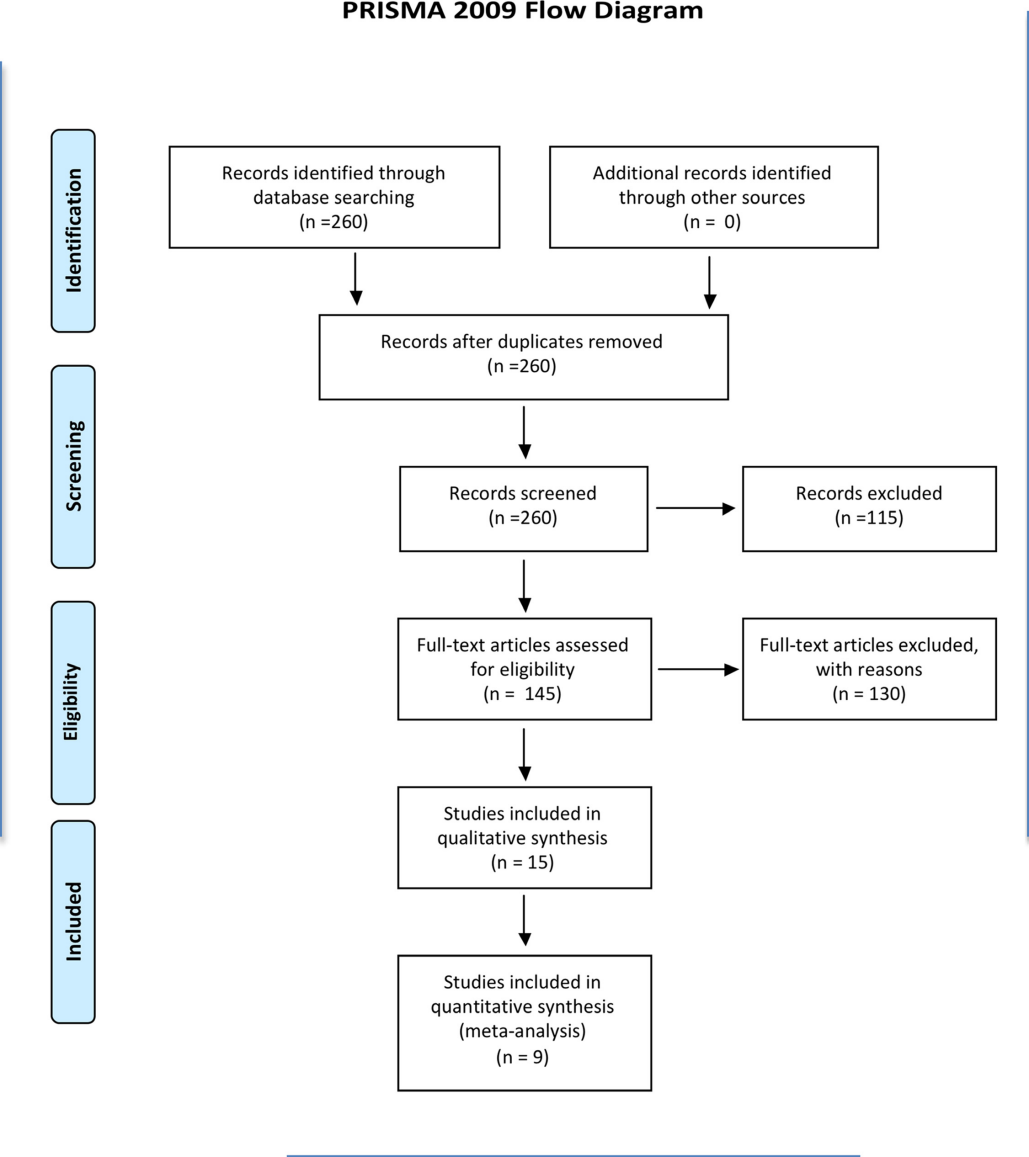
PRISMA flow diagram. The flow diagrams shows screening of literature and selection of studies for meta-analysis.

A robust search in PubMed (www.pubmed.com), Google Scholar (scholar.google.co.in) and ScienceDirect (www.sciencedirect.com) databases was conducted using the keywords such as ‘PCOS’, ‘MTHFR’, ‘677C>T’ polymorphism’ and ‘polycystic ovaries’ in various combinations. The search terms were kept broad to identify all relevant articles and the last search was performed on the 28^th^ of February 2015. The initial screening was done to look for *MTHFR* 677 C>T polymorphism data and the full text articles of relevant studies were obtained. The corresponding author was contacted in case a full text article was not accessible. Only fifteen studies containing data on 677 C>T polymorphism in PCOS could be identified (Fig 1).

#### Inclusion and exclusion criteria

Each article was thoroughly explored to extract the required information such as the purpose and design of study, data presentation, genotyping method used, inclusion and exclusion criteria adopted for recruiting cases and controls. The studies were further filtered to fit an inclusion criteria that (i) each trial was an independent case-control study, (ii) all the studies had a similar purpose of investigating the correlation of 677 C>T polymorphism with PCOS, (iii) enough information had been provided for the calculation of odds ratio (OR), (iv) SNP genotyping was done at the high resolution level, (v) selection of patients was done according to the standard and reliable diagnostic parameters. The studies not providing enough information (raw data) and those providing poor description of subject recruitment and analysis, were excluded.

#### Raw data extraction

Genotype data for the target polymorphism were extracted using the standard extraction form, and wherever necessary, the genotype frequencies were re-calculated to ensure homogeneous data presentation. The data were tabulated with specific details regarding the first author, year of publication, sample size, ethnicity of study population, and allele and genotype frequencies. Details required for standard reporting in systematic reviews and meta-analysis have been presented in PRISMA checklist (S1 Table) and meta-analysis on genetic association studies checklist (S2 Table). To ensure the correct representation, JC and SS performed data extraction using two independent forms and submitted it to the senior author of this article.

#### Quantitative data synthesis

Meta-analysis was conducted using the Comprehensive Meta Analysis software (version 2). We chose OR as the effect size to find PCOS risk associated with the rare genotypes (CT and TT). Raw data for each study were fed into software for calculation of the respective OR and p value. Heterogeneity test was performed using the chi-square based ‘Q’ test suggested by Cochran [33] and the inconsistency value (I^2^) given by Higgins [34]. In the presence of heterogeneity, random effects model was used while in the case of homogeneity, fixed effect model was used. An analysis model was chosen on the basis of the spread (distribution) of effect sizes and the level of heterogeneity. Since the test for heterogeneity suffers from low power, p values less than 0.10 were considered to be statistically significant. High-resolution plots were generated corresponding to OR and CI at 95% for fixed as well as random effects models. To check how the OR changed with subsequent addition of studies, a cumulative analysis was performed. Analyses using recessive and co-dominant models were also undertaken to thoroughly investigate the relationship between 677 C>T substitution and PCOS.

The data from each study were tested for fitness in the Hardy-Weinberg equilibrium using the chi-square method. The impact of assumptions on the robustness of inference was judged by sensitivity analysis. Publication bias was assessed using the funnel plot of precision by log odds ratio and calculated quantitatively using various statistical tools, such as Egger’s regression intercept, Duvall and Tweedie trim and fill procedure, Classic fail-safe ‘N’ and Orwin fail-safe ‘N’. Funnel plot was generated for the picturesque analysis of publication bias.

### Trial sequential analysis (TSA)

A meta-analysis is prone to systematic (bias) or random errors (play of chance) due to dispersed data and repeated significance testing. Bias from trials with low methodological quality, publication bias and small trial bias may result in false P-value. Therefore, we used trial sequential analysis (TSA) for the calculation of required information size (number of samples) for reliability of meta-analysis [35]. Some previous studies have shown that the outcomes of TSA are more reliable than those of the traditional meta-analysis [36–38]. We calculated the required information size by considering an overall type–I error of 5% and type-II error of 20%. TSA plotted a two-sided graph where red straight lines indicate significance boundaries of the conventional meta-analysis, the blue line shows cumulative Z-score, and red lines sloping inwards represent trial sequential monitoring boundaries with adjusted P-values.

## Results

### Clinical data

A comparative account of clinical data of patients falling in the hyper- and normo-androgenic groups is presented in Table 1. Almost all clinically relevant measures were significantly elevated in the hyper-androgenic group in comparison to the normo-androgenic group. The clinical values were also compared between patients with wild type (CC) and mutant (CT+TT) genotypes. Cross-classification analysis of hyper- or normo-androgenism versus genotypes (CC and CT+TT) did not show any significant association between the two (Table 2). The average homocysteine level in the cases (15.51 μmol/L, SD = 2.89) was not significantly different (P = 0.64) from the controls (15.89 μmol/L, SD = 2.23).

**Table 1:** Distribution and comparison of various clinical parameters between hyper- and normo-androgenic groups.

Clinical Parameters	Hyperandrogenism (N = 104) (Mean±SD)	Normoandrogenism (N = 157) (Mean±SD)	Comparison (P value)	Inference
Age (years)	29.17±5.15	30.11±5.47	0.3337	The mean age in hyperandrogenism is lower than the mean age in normoandrogenism.
Height (cm)	151.97±2.83	151.46±3.05	0.2699	The mean height in hyperandrogenism is almost similar to the mean height in normoandrogenism.
Weight (kg)	84.59±11.00	66.81±10.32	0.0001	The mean weight in hyperandrogenism is higher than the mean weight in normoandrogenism.
FSH (mIU)	5.98±1.22	5.56±1.04	0.0177	The mean FSH level in hyperandrogenism is significantly higher than the mean FSH level in normoandrogenism.
LH (mIU)	10.58±2.24	7.81±1.85	0.0001	The mean LH level in hyperandrogenism is higher than the mean LH level in normoandrogenism.
Random Blood Sugar (mg/ml)	162.8±41.51	114.06±35.30	0.0001	The mean random blood sugar level in hyperandrogenism is higher than the mean random blood sugar level in normoandrogenism.
LH/FSH	1.84±0.61	1.42±0.5	0.0001	The mean ratio of LH/FSH in hyperandrogenism is significantly higher than the mean ratio of LH/FSH in normoandrogenism.

**Table 2:** Distribution and comparison of various clinical parameters between *MTHFR* genotypes.

Clinical Parameters	Mutant (CT+TT) (Mean±SD)	Wild type (CC) (Mean±SD)	Comparison (P- value)	Inference
Age (years)	30.29±5.08	29.4±5.40	0.44	The mean age in Mutant genotype is similar than the mean age in Wild genotype.
Height (cm)	151.6±2.84	151.82±2.95	0.69	The mean height in Mutant genotype is almost similar to the mean height in Wild genotype.
Weight (kg)	76.26±13.3	76.09±14.09	0.95	The mean weight in Mutant genotype is similar than the mean weight in Wild genotype.
FSH (mIU)	5.37±1.14	5.89±1.13	0.01	The mean FSH level in CC genotype is significantly higher than the mutant genotype group.
LH (mIU)	9.22±2.51	9.28±2.49	0.91	The mean LH level in Mutant genotype is almost similar than the mean LH level in Wild genotype.
Random Blood Sugar (mg/ml)	154.37±55.57	135.66±41.68	0.03	The mean random blood sugar level in Mutant genotype is higher than the mean random blood sugar level in Wild genotype.
BMI	33.31±5.75	33.34±6.05	0.98	The mean BMI in Mutant genotype is almost similar than the mean BMI in Wild genotype.
LH/FSH	1.79±0.59	1.59±0.59	0.09	The mean ratio of LH/FSH in Mutant genotype is similar than the mean ratio of LH/FSH in Wild genotype.

### Case-control study

The genotype data for the control group fitted well in the Hardy Weinberg equilibrium (Exact test, p = 1.00). No significant association between 677C>T substitution and PCOS was observed in the pooled data of all cases versus controls (OR = 1.11, 95% CI = 0.71–1.72, P = 0.66). Similarly, in discrete analysis for each group (Indo-European and Dravidian), the p values remained statistically non-significant for the differences between cases and controls in each group (Indo-European p = 1, Dravidian p = 0.70). As mentioned above, we sub-grouped Dravidian patients on the basis of BMI; however, no difference was seen in genotype comparison of obese patients versus controls (P = 0.54), non-obese patients versus controls (P = 1.0), and obese PCOS versus non-obese PCOS patients (P = 0.68).

### Meta-analysis

#### Evaluation of studies

Following the above-mentioned search criteria, we could retrieve fifteen studies (Fig 1). Out of this, only nine met the inclusion criteria, and the remaining six were excluded because they were either case reports/reviews or were not case-control studies. The study by Palep-Singh et al. analyzed the polymorphism in two ethnically different groups; therefore, it was considered as two data sets. In the study by Idali et al., the two patient groups were merged because the only difference between them was the criteria followed for the diagnosis of PCOS i.e. either by Rotterdam Revised 2003 or by ultrasound. A total of 960 cases and 1028 controls, including those from the present case-control study, were included in the pooled analysis (Table 3).

**Table 3:** Case-control data included in the meta-analysis.

Study	Country	Race	Cases				Controls			
			Total	CC	CT	TT	Total	CC	CT	TT
Glueck et al. (1999b)	USA	Caucasian	**41**	14	23	4	**234**	119	89	26
Sills et al. (2001)	USA	Caucasian	**36**	25	9	2	**18**	8	9	1
Tsanadis et al. (2002)	Greece	Caucasian	**30**	12	14	4	**45**	20	19	6
Orio et al. (2003)	Italy	Caucasian	**70**	16	41	13	**70**	17	38	15
Palep-Singh et al. (2007)	UK	Caucasian	**25**	11	12	2	**16**	10	5	1
Palep-Singh et al. (2007)	UK	Asian	**21**	14	7	0	**9**	9	0	0
Choi et al. (2009)	Korea	Asian	**227**	67	125	35	**115**	33	67	15
Karadeniz et al. (2010)	Turkey	Turkey	**86**	15	65	6	**70**	35	28	7
Jain et al. (2012)	India	Asian	**92**	76	16	0	**95**	82	13	0
Idali et al. (2012)	Iran	Asian	**71**	36	31	4	**100**	66	25	9
Present study (2014) Combined	India	Asian	**261**	209	49	3	**256**	209	45	2
Present study (2014) Indo-European Population (IE)	India	Asian	**93**	77	16	0	**100**	83	16	1
Present study (2014) Dravidian Population (D)	India	Asian	**168**	132	33	3	**156**	126	29	1

#### Heterogeneity analysis and selection of analysis model

The effect sizes were spread and appeared to be the part of a normal distribution including an infinite number of studies. This favored the selection of random effects analysis model. Further, the ‘I^2^’ value of more than 50% for between studies comparison showed the presence of true heterogeneity (P_heterogeneity_ = 0.01, Q = 23.17, I^2^ = 56.84, Tau square = 0.18, SE = 0.15,var = 0.02). This further supported the choice of enrolling random effects model for the overall inference. However, the results of both the models (fixed and random) have been presented for reference.

#### Pooled estimate of effect size

**Fig 2:**
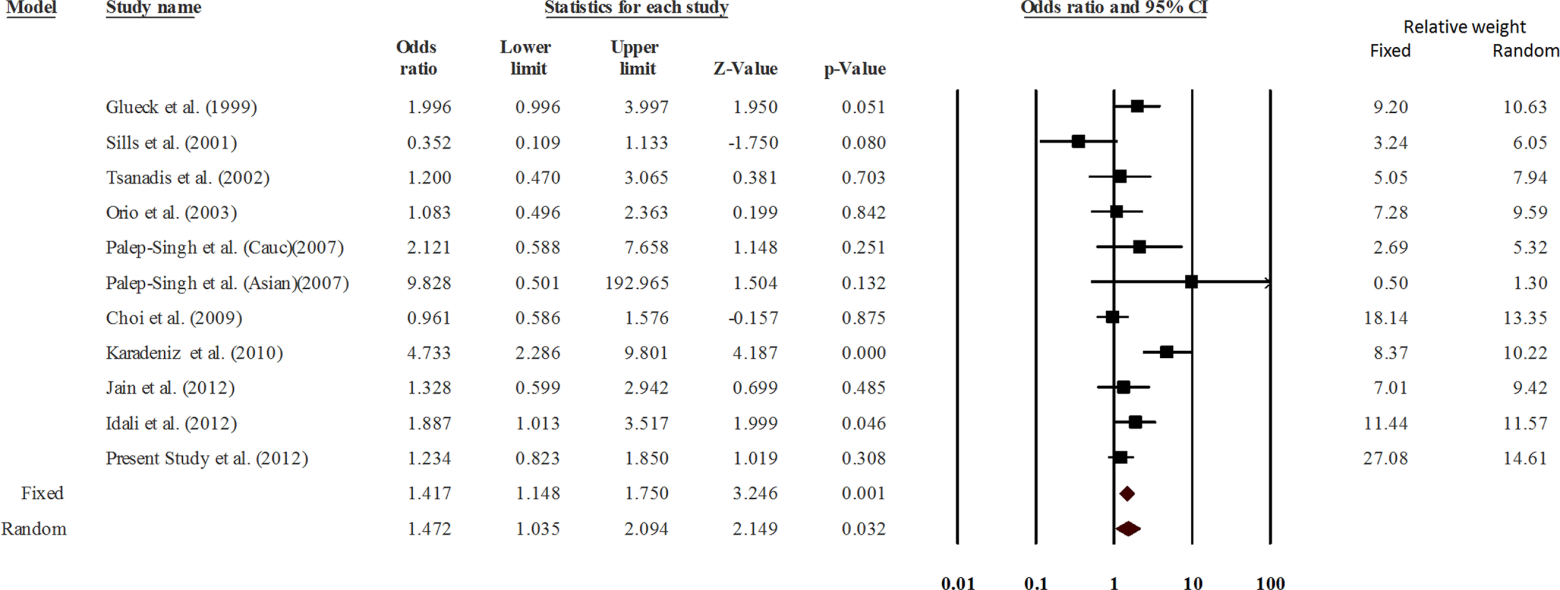
Meta-analysis. Forest plot showing the odds ratio, p value and direction of association between MTHFR 677 C>T polymorphism and PCOS. The Z value shows the degree and direction of the relationship, whereas the P value shows significance of the relationship. The horizontal bar shows the range of OR with a square in the centre, size of the latter is directly proportional to the weight given to each study. The direction of projection of the horizontal bar shows the direction of association.

**Fig 3:**
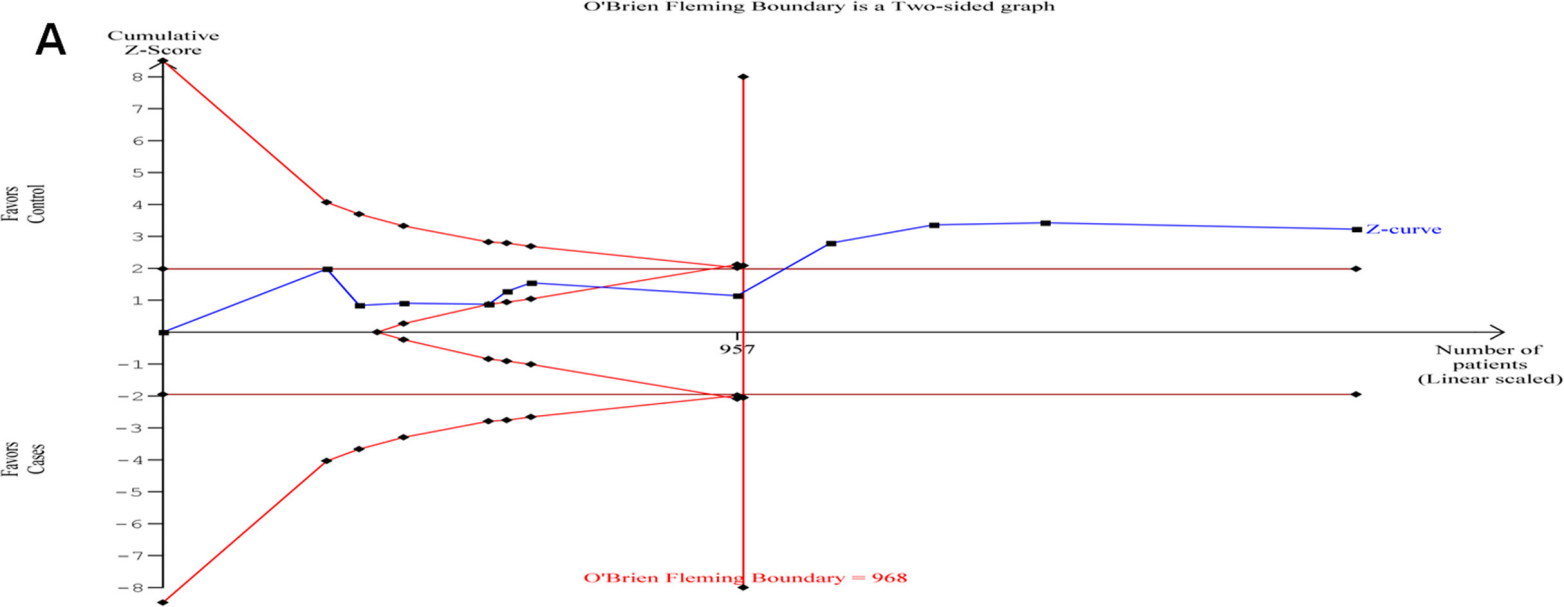
Trail sequence analysis of the studies included in the meta-analysis.

Analyses using both fixed effect and random effects models were undertaken with a priori preference for random effects model due to the expected heterogeneity. Analysis using the dominant model of analysis suggested a significant association of the substitution (CT + TT) with PCOS (p = 0.032, OR = 1.472, 95% CI = 1.035–2.094) (Fig 2). Outcomes of the TSA were concordant with results of the conventional meta-analysis and revealed that 677C>T polymorphism was significantly associated with PCOS risk. Moreover, it also revealed that enough number of samples and studies were included in the meta-analysis to reach a concrete conclusion as the number of samples crossed the O’Brien-Fleming boundary (Fig 3). The cumulative analysis did not show any significant trend in the p value up to the year 2009; however, beyond Choi et al. (2009), the p value showed a consistent decrease. Analysis using other genetic models did not show association between 677 C>T substitution and PCOS (Table 4).

**Table 4:** Results of meta-analysis (random effects model) using different analysis models.

Model		P-value	95% CI	Lower Limit	Upper Limit
Dominant (CC vs CT+TT)		0.032	1.472	1.035	2.094
Recessive (CT+CC vs TT)		0.389	0.841	0.566	1.248
Co-dominant	CC vs. CT	0.115	0.675	0.414	1.101
	CC vs. TT	0.520	1.183	0.709	1.975
	CT vs. TT	0.070	1.405	0.973	2.029

**Fig 4:**
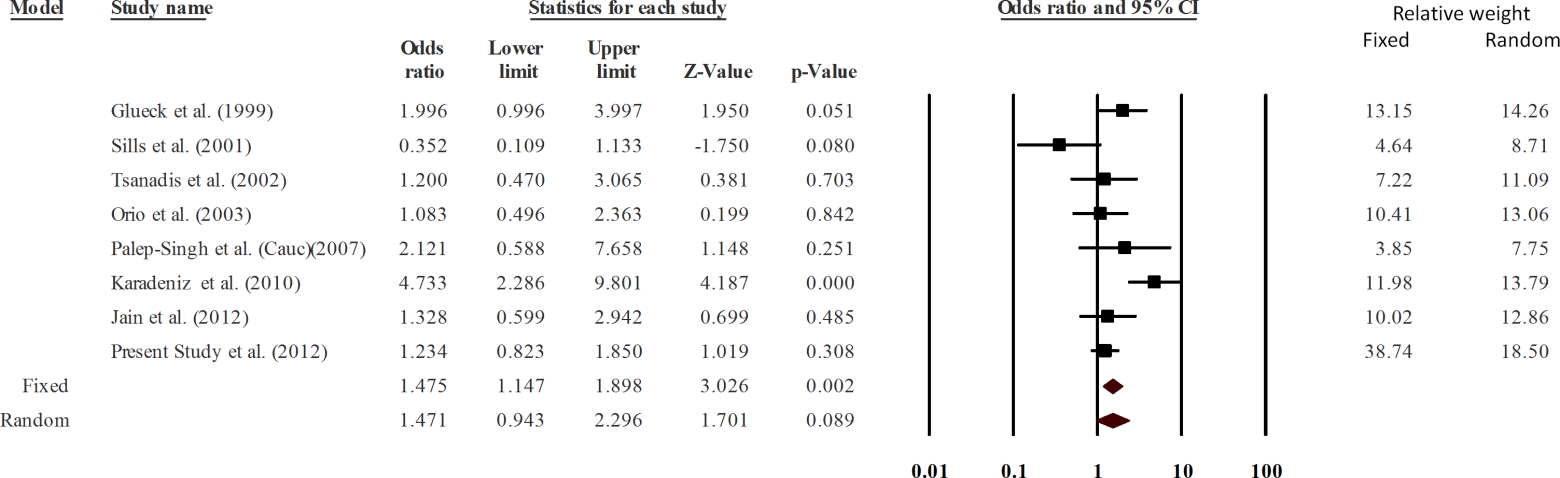
Sensitivity analysis excluding the studies not fitting the Hardy Weinberg equilibrium.

The data of all studies, except that of the Asian group of Palep-Singh et al (2007), Choi et al. (2003) and Idali et al. (2012), fitted well in the Hardy Weinberg equilibrium. Therefore, a sensitivity analysis was conducted after excluding the above three studies. The data were still significantly heterogeneous (Q = 18.26, p = 0.011, I^2^ = 61.67, Tau square = 0.24, SE = 0.22, var = 0.048) and the p value turned non-significant (random effects P = 0.089) (Fig 4). Therefore, we concluded that the studies not following the Hardy Weinberg equilibrium affected the results of meta-analysis significantly. A careful look into the pooled data showed that the association was ethnic specific. Among all the studies pooled in this analysis, only one study on a Turkish population reported a highly significant association of 677C>T substitution with PCOS [18]. We undertook a pooled analysis after its exclusion, which suggested no association of 677 C>T substitution with PCOS [P = 0.071; OR = 1.28 (0.98–1.67)]. Therefore, 677 C>T polymorphism seems to be irrelevant to PCOS in most of the populations studied so far, except in the case of Turkish populations.

#### Publication bias

**Fig 5:**
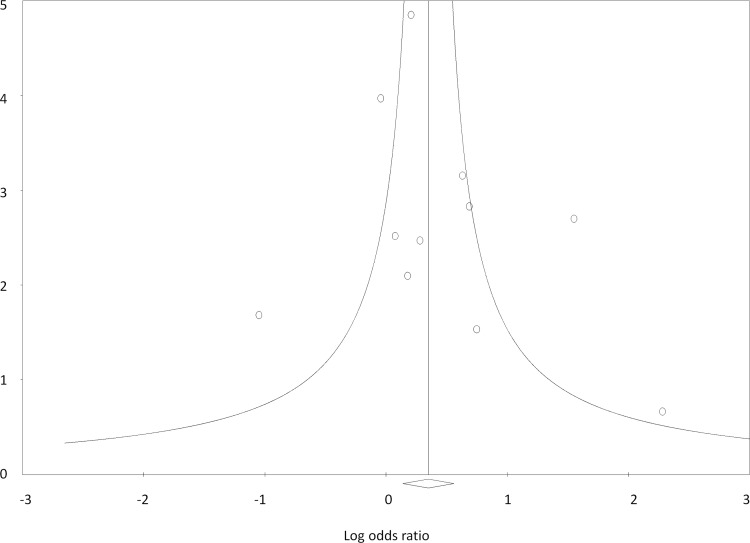
Funnel plot of precision by log odds ratio showing the absence of publication bias.

Egger’s test showed no evidence of publication bias (B_0_ = 0.75, SE = 1.21, 95% CI = -1.98–3.48, two tailed p value = 0.55). Classic fail-safe ‘N’ value of 22 and Orwin’s fail-safe ‘N’ value of 30, suggested 2.2 and 3 studies, respectively, missing from the analysis for every study included. Since the possibility of missing so many studies is negligible, the existence of no bias seems more plausible. Picturesque view of the funnel plot also showed almost symmetrical distribution of the studies, further ruling out publication bias (Fig 5).

## Discussion

Glueck et al. (1999) analyzed *MTHFR* 677C>T for the first time in 41 PCOS cases and 234 control women, and found no significant difference in the distribution of heterozygous and homozygous genotypes between cases and controls (P>0.09) [22]. Later, a pilot study (Cases = 36, Controls = 18) also suggested no association between 677 C>T substitution and PCOS risk [19]. Yet another work on 30 cases and 45 controls suggested no significant association between this polymorphism and PCOS [21]. Similarly, Orio et al. (2003) after investigation on 70 Italian patients and an equal number of controls reported no correlation between 677 C>T genotypes and PCOS risk [23]. Palep-Singh et al. (2007) investigated the association between this substitution and PCOS in Asian (21 cases and 9 controls) and Caucasian study groups (25 cases and 16 controls) and reported no significant correlation between the *MTHFR* genotypes and PCOS [24]. Due to small sample sizes, data generated by the above studies lacked the power of independent representation. Nevertheless, it is interesting to note that all the initial studies reported a lack of correlation between the above polymorphism and PCOS risk. Two genome wide association studies (GWAS) on PCOS have been published; however, none of them included 677 C>T substitution in the analysis [39,40].

We observed no significant correlation between the *MTHFR* genotype and PCOS risk in Indian populations. The two ethnically different groups provided us the opportunity to also test the effect of ethnicity on PCOS risk. The north Indian populations have an Indo-European ethnic affinity and are strikingly different from the Dravidian populations [41]. There was no association either in any of the two study groups or in the collective analysis. Classification of the patients according to the BMI also failed to find any difference between lean/obese cases and controls. We have used the largest sample size till date and observed no correlation of 677 C>T genotypes with PCOS in Indian populations. Further, no significant difference in homocysteine level between cases and controls ruled out any significance of homocysteine measurement in PCOS cases. Another recent study also reported a lack of association between this polymorphism and PCOS risk in India [25].

At least three previous meta-analyses have analyzed 677 C>T polymorphism, two of which stated no correlation [27, 28] while one reported a significant association of the substitution with PCOS [26]. Bagos (2009) conducted a meta-analysis on six data sets published between 1999 and 2007 and reported no impact of 677 C>T substitution on PCOS risk [28]. A careful look into this meta-analysis brought forward the problems in data presentation since the values for homozygous rare and homozygous common genotypes had been interchanged for some of the studies [28]. Fu et al. (2014) conducted a meta-analysis on nine studies representing 638 cases and 759 controls [26]. The authors reported that *MTHFR* 677C>T polymorphism was a risk factor for PCOS in the dominant model and in CT vs TT comparison. In another meta-analysis on 607 PCOS and 663 controls from eight studies, Lee et al. (2014) reported that 677 C>T polymorphism was not a risk factor for PCOS [27]. However, none of the meta-analyses described above undertook a trial sequential analysis, which evaluates a meta-analysis for power and sample size. We conducted a meta-analysis on 960 cases and 1028 controls and observed a significant association of 677 C>T substitution with PCOS. Nevertheless, sensitivity analysis brought forward an interesting observation that the studies showing deviation from the Hardy Weinberg Equilibrium influenced the results of meta-analysis significantly. Exclusion of these studies suggested a lack of correlation between 677C>T substitution and PCOS.

Among the studies pooled in the analysis, only one study on a Turkish population reported a significant association of 677C>T substitution with PCOS. In order to analyze for its influence on meta-analysis, we undertook a pooled analysis after the exclusion of Karadeniz et al. 2010. The exclusion suggested no association of this polymorphism with PCOS. Therefore, 677 C>T polymorphism seems to be unrelated to PCOS in most of the populations studied so far, except in Turkish populations. This suggests the existence of sensitive studies that influenced the association of 677 C>T polymorphism with PCOS. Trial sequential analysis showed that the meta-analysis had enough sample size; however, the overall conclusion was strongly influenced by the sensitive studies. Therefore, further studies need to evaluate the association of this polymorphism with PCOS in Turkish populations. While genetic risk factors certainly affect PCOS risk, the end result may a complex outcome of an interplay between genetic and lifestyle factors such as insulin resistance, obesity and physical inactivity, which can further promote its occurrence [42]. Eventually, lifestyle modification programmes highlighting the behavioural management, dietary and exercise interventions have been effective in the general population, reducing the risk of diabetes and the metabolic syndrome, and have had some initial success in taming fertility upshots in PCOS [42].

In conclusion, MTHFR 677 C>T genotype does not influence the risk of PCOS in the study populations and most other populations. Further, the polymorphism is not differently distributed between obese and non-obese cases, ruling out its correlation with obesity accompanied PCOS. A direct correlation of 677 C>T polymorphism with PCOS risk was ruled out in two ethnically different Indian populations. Though meta-analysis suggested 677 C>T substitution to be a PCOS risk factor, critical analysis of the pooled data suggested that the conclusion was significantly biased. TSA validated this observation for sufficient sample size and power. Therefore, we have now reached an adequate sample size for taking a decision regarding the clinical importance of 677C>T substitution in PCOS cases. Exclusion analysis suggested that the association in the meta-analysis was largely due to the study by Karadeniz et al. (2010) on a Turkish population. The association was skewed due to the studies that deviated from the Hardy Weinberg equilibrium. Therefore, the exclusion of sensitive studies mentioned above revealed that 677 substitution has no clinical importance in PCOS. If it affects PCOS risk, the association is likely to be ethnic-specific and further studies on various populations would pave the way for identification of ethnic-specific effects. Since the association in Turkish populations is reported by one study only, further studies on Turkish or ethnically similar populations are encouraged. Studies on ethnically divergent populations such as East Asians; particularly Chinese and Japanese, may provide new data; nevertheless, there is now sufficient evidence to conclude that MTHFR 677 C>T is not clinically important in PCOS in most of the populations.

## Supporting Information

S1 Table PRISMA checklist for meta-analysis. (DOC)

S2 Table Meta-analysis on genetic association studies checklist. (DOCX)
